# The Impact of Intracytoplasmic Sperm Injection in Non-Male Factor Infertility—A Critical Review

**DOI:** 10.3390/jcm10122616

**Published:** 2021-06-14

**Authors:** Tanya L. Glenn, Alex M. Kotlyar, David B. Seifer

**Affiliations:** Department of Obstetrics, Gynecology, and Reproductive Sciences, Section of Reproductive Endocrinology and Infertility, Yale University School of Medicine, New Haven, CT 06520, USA; alexander.kotlyar@yale.edu (A.M.K.); david.seifer@yale.edu (D.B.S.)

**Keywords:** ICSI, non-male factor infertility, overutilization, IVF, ART, outcomes, cost burden

## Abstract

Intracytoplasmic sperm injection (ICSI) was originally designed to overcome barriers due to male factor infertility. However, a surveillance study found that ICSI use in non-male factor infertility increased from 15.4% to 66.9% between 1996 and 2012. Numerous studies have investigated fertilization rate, total fertilization failure, and live birth rate per cycle (LBR), comparing the use of ICSI versus conventional in vitro fertilization (IVF) for non-male factor infertility. The overwhelming conclusion shows no increase in fertilization rate or LBR per cycle with the use of ICSI for non-male factor infertility. The overuse of ICSI is likely related to the desire to avoid a higher rate of total fertilization failure in IVF. However, data supporting the benefit of using ICSI for non-male factor infertility is lacking, and 33 couples would need to be treated with ICSI unnecessarily to avoid one case of total fertilization failure. Such practice increases the cost to the patient, increases the burden on embryologist’s time, and is a misapplication of resources. Additionally, there remains conflicting data regarding the safety of offspring conceived by ICSI and potential damage to the oocyte. Thus, the use of ICSI should be limited to those with male factor infertility or a history of total fertilization factor infertility due to uncertainties of potential adverse impact and lack of proven benefit in non-male factor infertility.

## 1. Introduction

The emergence of intracytoplasmic sperm injection (ICSI) in the early 1990s, just a few years after the advent of in vitro fertilization (IVF), transformed the treatment of male infertility. Its original purpose was to serve those with severe male factor infertility (MF) or those with a history of unexplained total fertilization failure (TFF). Shortly following the introduction of ICSI, a study analyzed fertilization rates (FR) and pregnancy rates (PR) in individuals with severe MF or history of multiple TFF while stratifying by the severity of MF and sperm source [1]. They noted a drastic improvement in PR in the 227 couples within the study that was not altered by sperm source or concentration. Over half of these couples had previously experienced a TFF, but with ICSI, the average FR was 63.9%, and only two couples (approximately 0.9%) had TFF [1]. Yet, these improvements were not seen in all types of MF, as Keegan et al. in 2007 determined no differences in FR, TFF, or LBR per cycle in men with normal sperm compared to those with teratozoospermia in either IVF or ICSI cycles [2]. However, current use has greatly surpassed its original intended indications. Recent statistics show an increase in ICSI use from 36.4% in 1995 to over 76% in 2012, with the greatest increase in non-male factor infertility (NMF) from 15.4% to 66.9% [3]. The most recent practice committee opinion by the American Society of Reproductive Medicine (ASRM) in 2020 concerning the use of ICSI for NMF indicated that NMF counted for anywhere between 68%–72% of the use of ICSI [4]. Prior to utilizing ICSI, it is important for clinicians to understand the data behind the use of ICSI for NMF, potential harm to offspring, cost burden to patient and laboratory time, as well as potential harm to oocytes or embryos.

## 2. Methods

A medical literature search was performed in Pubmed and The Cochrane Library. Phrases used in the search were suited for each individual database and included “Intracytoplasmic sperm injection” OR “ICSI” AND “non-male factor”. Our search period spanned from 1994–2021. Three hundred thirty-eight articles were found. These articles were then assessed for relevance and quality. Only studies published in English were included 40 of these studies were included as part of this review.

The primary outcomes of this review were to determine if the use of ICSI for NMF improved reproductive outcomes, specifically, live birth rate (LBR), fertilization rate (FR), and total fertilization failure (TFF). Additionally, the potential impact on offspring, associated costs of ICSI, and impact on oocytes or embryos. Articles were selected as relevant if they were: practice guidelines, retrospective reviews, retrospective cohort studies, observational studies, randomized control trials (RCTs), prospective studies, systematic reviews, or meta-analyses that evaluated ICSI in the setting of NMF. Studies were excluded if they were (1) case reports, editorials, abstracts, non-systematic reviews, (2) did not include NMF or the use of ICSI, or (3) did not include one of the following: LBR, FR, TFF, birth defects, costs, or impact on oocytes.

## 3. IVF Outcomes for NMF

The use of ICSI has been proposed as a universal protocol in some clinics, while other clinics are more selective in utilizing ICSI in certain subgroups of infertility other than MF or TFF. Some of these subgroups include poor ovarian reserve (POR), diminished ovarian reserve (DOR), poor oocyte quality, advanced maternal age, low oocyte yield, unexplained infertility, or mild MF. Unfortunately, there have been few RCTs performed examining this topic, and even fewer that have specifically analyzed patient-driven outcomes, namely LBR per cycle. Mainly due to the nonrandomized design of many studies, there is an inherent high risk of bias.

### 3.1. Unselected Patient

The misapplication of ICSI beyond severe MF was notable early in its conception that went far beyond the designated patient population. The first Cochrane Review of ICSI for NMF was performed in 1999, with an update in 2003. Yet, no RCT at this time evaluated LBR [5]. During that same time, Khamsi et al. reviewed four articles that performed ICSI and IVF on sibling oocytes for NMF. They found that half of the studies showed improvement in FR and a reduction in TFF in three of four of the studies with the use of ICSI [6]. It is important to note that only MII oocytes were exposed to ICSI versus all oocytes for IVF, leading to likely selection bias when reporting FR. Additionally, the TFF rates in each study were unexpectedly high (22.7%, 11.4%, and 6.8%), which may be reflective of older laboratory technology [6].

An unselected prospective controlled study from 2001 for 35 patients examined any patients with NMF and found higher FR with ICSI (57.2% vs. 71.3%, *p* = 0.005); however, no pregnancy outcomes were reported [7]. Another early study showed a slightly decreased TFF rate when ICSI was used indiscriminately; however, they calculated that 33 couples would need to be treated unnecessarily to prevent one TFF [8]. In contrast to these studies, a prospective cohort of 486 patients with 696 cycles found no difference in FR, TFF, or LBR over a 2 year period in 2007 [9]. Two retrospective reviews showed slightly differing results from each other. One was an analysis of the Latin America Registry of Assisted Reproductive Technology (ART) of nearly 50,000 ART cycles from 2012 to 2014 and determined that although FR did not differ between the two groups, LBR was higher (22.99% vs. 28.76%, *p* < 0.001) and TFF was actually lower (4.49% vs. 3.37%, *p* < 0.001) in the IVF group [10]. The other retrospective analysis from 2009–2015 with over 3000 patients found that FR and LBR per cycle were higher in IVF than ICSI (67.1% vs. 62.3% *p* < 0.0001, 17.22% vs. 13.2% *p* < 0.001, respectively). They stated that “one less pregnancy in every 15 cycles where ICSI was used without an indication” [11]. One study specifically attempted to look at the use of ICSI in NMF to help reduce TFF and improve embryo quality [12]. They found in their cohort of ICSI (advanced age, history of poor fertilization, repetitive implantation failure, endometriomas, low oocyte quality, and low oocyte yield; *n* = 142) that the TFF did not vary, but normal FR rate did improve with ICSI (83.4% and 79.1%, *p* = 0.04, respectively) [12]. Most recently, a 2020 RCT assessing NMF with sibling oocytes did find a slightly higher TFF in IVF cases (10 cases); however, they did not analyze LBR [13]. Please see Table 1.

Yet, it is important to realize that their study groups were vastly different and confounded by the age of male and female partners, the number of oocytes retrieved, and that ICSI only utilizes mature oocytes, while IVF includes all oocytes (immature and mature) during insemination. They also did not identify the reasons/indications for using ICSI; thus, their results were less generalizable [12]. The disparity in results is likely reflective of inherent bias embedded in retrospective or nonrandomized prospective studies, as well as differences in patient and IVF center-driven factors. Additionally, caution should be used applying data from the early 2000s, given the significant clinical and laboratory advancements in ART in the last 20 years.

More recent studies comparing the use of ICSI in the general population versus IVF include several retrospective cohorts analyzing LBR. One published in 2018 from Australia examined the Victorian database of 15,000 cycles with no difference reported in LBR per cycle between IVF and ICSI for NMF [3]. The second study published in 2019 from the United States (US) at a single institution also showed no significant difference in LBR per transfer but an increase in FR [20]. Zagadailov et al. examined different geographical regions throughout the US to determine ICSI utilization, LBR per cycle, and MF diagnosis [27]. They noted a striking difference in ICSI utilization across the United States, with the highest rates in the Front Range (including the areas of Albuquerque, Cheyenne, Colorado Springs, Denver, Pueblo, Salt Lake City) and Gulf Coast, and lowest rates of ICSI utilization in the Northeast and Florida [27]. However, having a higher ICSI rate was not associated with increased rates of MF nor a strong association with increased LBR per cycle [27]. Thus, pointing out that ICSI utilization had more to do with where geographically a clinic was located than it had to do with the etiology of infertility. Furthermore, retrospective analysis of over 20,000 patients found no difference in LBR per cycle between the IVF and ICSI, even when including mild and moderate MF [24]. Please see Table 1.

### 3.2. Advanced Maternal Age

As stated earlier, clinicians often cite reasons other than those recommended by ASRM for the use of ICSI, particularly maternal age, but what do studies for these etiologies of infertility show [28]? In 2017, a retrospective study of women 40–43 who underwent either IVF or ICSI for NMF also showed no difference in FR, TFF, or LBR, and that the IVF group had more embryos to freeze [18]. Yet, the authors did note that the ICSI group had undergone more IVF cycles previously, with 58% of the IVF group having their first cycle, while only 33.2% of the ICSI group was undergoing their first cycle [18]. In contrast with this information, a recent retrospective cohort study in 2019 of only 52 women >35 years old with 6+ oocytes split between IVF and ICSI [22]. They examined FR and embryo quality and noted a non-significant increase in both with the use of ICSI. Importantly, the study did remark that the more mature oocytes may have been selected by the embryologist preferentially for ICSI, thus introducing selection bias with the results skewing in favor of ICSI [22]. Liu et al.’s retrospective cohort of 644 patients from 2011–2016, women 40–43 years old in their first IVF cycle, with the same protocol and low egg yield, found an increase in cumulative LBR per cycle for the IVF group after adjusting for primary infertility (14.6% vs. 5.6%, *p* < 0.05) [19]. In a similar fashion, a 10-year retrospective review of women 35 years old or greater showed an increase in LBR per cycle in women who received IVF compared to ICSI (aOR 2.64, *p* < 0.0001) [26].

One theory that supported the use of ICSI for NMF in older women was the speculation that the zona pellucida hardened with age [29]. The majority of studies have highlighted the complete lack of high-quality RCTs in this area and the high risk of selection bias introduced by the less-than-optimal study design. Fortunately, early in 2021, an RCT attempted to overcome these flawed study designs by assessing advanced maternal age (39 years and older) and the use of ICSI [23]. In this study, oocytes were randomized to IVF/ICSI by ovary through computer-generated randomization, therefore eliminating selection bias by the embryologist to choose which oocyte underwent IVF or ICSI. They noted no difference in FR between the two groups from an appropriately powered study, and one TFF was seen in both groups [23]. Unfortunately, LBR was not included. Overall, these studies do not support the indiscriminate use of ICSI for NMF due to maternal age. However, there remains the question of what maternal ‘age’ we should consider to be advanced? Each study reviewed utilized a different age cut-off, which should motivate clinicians to establish a more standardized term and encourage researchers to perform better-controlled studies utilizing sister oocytes. Please see Table 1.

### 3.3. Poor Ovarian Reserve

The term POR has been used inconsistently in the ART literature. Per the Bologna criteria, POR includes women who meet two of the following criteria: 40 years old or older, have another risk factor for POR, had a previous IVF cycle that only achieved three or fewer oocytes, or had an abnormal ovarian reserve test (i.e., low antral follicle count < 5–7, or an AMH < 0.5–1.1 ng/mL) [30]. However, since this definition was not established until 2011, many earlier studies utilized a variety of different definitions for POR. Thus, for the sake of this discussion, we will combine all studies that include patients labeled as POR, low oocyte yield, or DOR. Despite the variations in definitions, all studies exemplified the limitation of what was stated earlier: a lack of proof that ICSI improved LBR or FR. A retrospective study from 1991–2016 using the national Human Fertilisation and Embryology Authority (HEFA) database singled out over 60,000 cycles meeting criteria for POR with autologous oocytes [25]. The LBR per cycle was equal between the two groups (IVF: 12.2% and ICSI: 12.4%), as was TFF [25]. A 2015 study also specifically utilized the Bologna criteria to determine if ICSI is beneficial in those with POR and NMF. This retrospective study of 243 patients had a primary outcome of LBR per cycle with secondary outcomes to include FR, in which they noted no differences [16].

One of the larger studies performed across 15 centers in Europe analyzed nearly 5000 cycles of NMF undergoing their first IVF cycle with an antagonist protocol divided the groups by ovarian response (poor, suboptimal, normal, or high responders) [21]. Although ICSI was overwhelmingly utilized in this cohort (4227 versus 664), no difference in FR or LBR was noted between the two types of inseminations no matter what the ovarian response [21]. A recent study in 2020 in patients who had low numbers of oocytes retrieved (<6 oocytes) FR was equivalent between IVF and ICSI. They also singled out DOR patients, yet this SART database review actually showed a statistically significant decrease in LBR per cycle with the use of ICSI (20.4% versus 21.9%) [15]. Additionally, as only mature oocytes are exposed to sperm with the use of ICSI, the FR per oocyte retrieved was often higher with the use of conventional IVF [15]. In this same study, 5% of couples who used IVF and 2% of those using ICSI experienced TFF; thus, 33 ICSI procedures would have needed to be completed to prevent 1 case of TFF [15]. Thus, not only is the use of ICSI not beneficial for POR or DOR, but some studies show an improvement in LBR with the use of IVF when used in the context of NMF. There is also an enormous cost burden on laboratories to perform ICSI for all women with POR/DOR to prevent one case of TFF, and there is concern that this could result in a decrease in the number of useable embryos with ICSI. Please see Table 1.

### 3.4. Mild Male Factor

ICSI’s original intentions were to enable those individuals/couples with severe MF that required surgical sperm extraction to have biological children. However, mild MF was not part of this original indication, although today, any subtle MF often leads clinicians and/or embryologists to lean towards ICSI. In a sub-analysis of an RCT from 2001, LBR was not evaluated; however, no difference in TFF was seen between groups [8]. In 2002, a meta-analysis reported that if the standard insemination concentration of 0.2 × 10^6^/mL was utilized in those with mild MF, FR was improved if ICSI was used. Yet, this was no longer applicable if a higher concentration of 0.8 × 10^6^/mL was utilized [14]. However, when comparing these results to more recent results, Esteves et al. noted that those with only mild MF, 5 × 10^6^/mL to <15 × 10^6^/mL and <32% forward motility, IVF, and ICSI had no difference in FR or LBR [31]. Once again, this data highlights the inadequacy of ICSI to improve outcomes when used indiscriminately compared to IVF.

### 3.5. Tubal Factor

Although tubal factor seems like an unlikely reason for ICSI, this diagnosis is still often used. Grimstad et al. reported that from 2004–2008 ICSI was utilized in 50% of women who had a diagnosis of tubal factor infertility only [17]. Their retrospective review of 7000 cycles from the SART database of only tubal factor infertility showed a non-significant increase in FR in ICSI, but a decreased adjusted odds ratio (aOR) for LBR per cycle with the use of ICSI (0.77, (CI 0.69–0.85)) [17]. Notably, as this is a retrospective review of the SART database and not all confounding factors could be analyzed but indicates that lower LBR can result when using ICSI in contrast to IVF for NMF [17]. Please see Table 1.

## 4. ICSI and Effects on Offspring

In addition to the discussion concerning pregnancy outcomes, the safety of ICSI should also be taken into consideration. Currently, there are conflicting data regarding the safety of ICSI for NMF, especially since larger studies often contain both MF and NMF. Studies do indicate that couples with infertility who utilize IVF/ICSI have a higher rate of fetal anomalies than their fertile counterparts. However, in couples who struggle with fertility IVF may be the best option, and for those with severe MF or a history of TFF, ICSI is the only option. Although for patients with NMF infertility, the risk of ICSI is not outweighed by any real benefit.

Here we consider the safety of IVF and ICSI, regardless of the diagnosis of infertility. The revised Cochrane Review in 2003 highlights the complex nature of this issue, as some studies demonstrated an increase in major congenital birth defects, while others noted an increase in paternally inherited chromosomal aberrations, as well as aneuploidy and structural changes [5]. Another review approached the potential for epigenetic changes with ICSI, increasing the risk of specific diseases, namely Beckwith–Wiedemann Syndrome, Silver-Russel Syndrome, and Angelman Syndrome, yet the authors point out that some studies show how rare these imprinting disorders are, with a prevalence <1% even in ICSI [32]. Additionally, cord blood analysis of children born spontaneously, through IVF, or through ICSI did show a decrease in methylation rates of those utilizing ICSI; however, the changes were small, and the effect on the offspring is unknown [33]. Another study corroborated this by observing a reduction in methylation within the placenta of ICSI pregnancies [34]. A large retrospective study in 2008 utilizing birth records in South Australia identified over 6000 births from ART with an increase in birth defects in patients who utilized ICSI [35]. When combined, IVF and ICSI were associated with an increased risk of birth defects with an aOR of 1.24 (CI 1.09, 1.41), but the aOR of only IVF was not significant [35]. Furthermore, when comparing IVF and ICSI, IVF had a reduced risk of birth defects compared to ICSI in fresh cycles with an OR of 0.68 (CI 0.53, 0.87) [35]. This was corroborated by a 2012 meta-analysis of 56 studies comparing IVF and ICSI to naturally conceived children, which showed a significantly increased risk of birth defects in both IVF and ICSI children. They also noted a greater increase in those with ICSI versus IVF that was non-significant. Their conclusion was that the most valid control group would have been naturally conceived children of infertile couples rather than couples who had no fertility problems [36]. This reflection is even more important when analyzing a Danish study in 2006 that recognized an increase in congenital malformations in couples who struggled with infertility, even if they did conceive naturally [31]. However, there is limited information about infertile couples who conceive without ART in order to compare IVF and ICSI for outcomes.

These results contrast with a systematic review published around the same time as the 2003 Cochrane Review of data from 4 studies of over 5000 children between 1988–2002. The review examined the most common birth defects: hypospadias, cleft lip/palate, cardiovascular, musculoskeletal, and neural tube defects. They noted no significant difference between ICSI and IVF in these areas [37]. Analyzing natural conception versus ICSI for NMF, no differences were seen in the karyotype abnormalities of miscarriages [38]. ASRM published a practice committee opinion in 2008 discussing the ramifications of ICSI. They determined that although the risk for congenital malformations is likely low, the intellectual or motor development of an offspring is uncertain, and there is an increased risk of sex chromosomal abnormalities. Whether this is due to the procedure or the underlying male factor is undetermined [39]. In early 2021, a large Society of Assisted Reproductive Technology Clinical Outcome Reporting System (SART CORS) database review from several states over 10 years analyzed the risk of birth defects separating out IVF, ICSI for MF, and ICSI for NMF compared to non-ART children, either through ovulation induction or natural conception [40]. Importantly, they also examined non-ART siblings. For singleton births, the risk for any non-chromosomal defect was increased in those who utilized IVF with aOR 1.18 (95% CI 1.05–1.27). However, those who utilized ICSI had a drastic increase in in non-chromosomal defect with and without MF, at an aOR of 1.30 (1.16,1.45) and 1.42 (1.28,1.57), respectively [40]. This study of over 20,000 ART children born with anomalies emphasized that ICSI is not without risks [40].

When taking a closer look at the impact of ICSI for NMF on motor or intellectual development, a clear picture is also difficult to obtain. Intellectual disability was highlighted in the Australian study that compared ART in general versus natural conception. Although they did not parse out the use of ICSI for NMF, they did see a higher rate of intellectual disability in those utilizing ICSI with an RR of 2.54 (95% CI 1.69–3.83) after an eight-year follow up [41]. In 2014 Kissin et al. examined ART and the risk of autism for offspring [42]. In order to have accurate data into young childhood, they restricted their analysis from 1997–2006 and children who had been born by ART in California with 5 years of follow-up. Among singleton pregnancies, the overall rate of autism was 0.8%; however, when ICSI was used, this was increased with an adjusted hazard risk ratio (aHRR) of 1.71. This increased risk remained significant even when the diagnosis was NMF [42]. As stated earlier, the concern for epigenetic changes induced by ICSI has been postulated by several studies, yet the clinical impact of these changes is unknown, and none of these studies analyzed specifically if ICSI for NMF still induced these changes.

Overall, there is a significant amount of selection bias, statistical heterogeneity, and variability in study design that is present when analyzing congenital birth defects and mental health [31]. There does seem to be an increase in congenital malformations in patients who experience infertility, but the precise differences between ICSI and IVF, specifically for NMF, are difficult to discern accurately. Some theories behind the potential increase in congenital malformations with the use of ICSI over IVF for MF are attributed to the manipulation of sperm, abnormalities within the sperm, and a change in the in vivo sex steroid hormone levels [31]. However, only the manipulation of sperm would be relevant for those utilizing ICSI for NMF. Overall, it is an area that requires a more intensive, standardized analysis. Until then, it is prudent to inform patients of the uncertainty surrounding the utilization of ICSI for NMF without any known benefit.

## 5. ICSI and Costs

Cost is another factor when considering the use of ICSI for purposes outside of those delineated in the 2020 ASRM guideline [28]. Several institutions estimated that the cost of ICSI added 5%–8% to the already high cost of IVF [20]. Multiple studies have also shown that clinics/physicians who reside in states with mandatory IVF coverage are more likely to adhere to the ASRM guidelines for when to utilize ICSI and pursue elective single embryo transfer [43,44]. One study retrospectively analyzed nearly 1.4 million IVF and ICSI cycles from 2000–2015 and noted that ICSI use increased dramatically in states without coverage (34.6% to 73.9% vs. 39.5% to 63.5%). Another showed that MF and ICSI rates had no correlation, despite the vast increase in ICSI use [43,45]. They also emphasized that this was most obvious in the context of unexplained infertility or the low number of oocytes retrieved. The authors hypothesized that clinics in non-mandated states have a financial conflict of interest and that clinics need to balance patient cost with the cost of success [43]. Others commented that mandated states are more likely to be selective in how they utilize laboratory resources [44].

Financial cost should not be the only important consideration, but also the embryologist’s time. In 2001, four British IVF centered randomized couples with NMF to IVF or ICSI and noted that the lab time required for each was over three times greater for the ICSI cases versus IVF and had a decrease in pregnancy rate [8]. They concluded that this was valuable time that the embryology team could have utilized on other patients’ care. Both added financial cost and additional labor costs of embryologist’s time eventually result in higher charges for a cycle of ART. Over time ART becomes less affordable and more inaccessible to those without insurance coverage leading to greater disparities in care.

## 6. ICSI and Impacts on the Oocyte

Finally, the potential risk to the oocyte should not be underestimated when performing nonessential ICSI. Studies have reported anywhere from a 5%–19% mechanical damage rate to oocytes during the ICSI procedure [20]. Ebner et al. specifically investigated procedural deviations from the standard ICSI technique from over 2200 procedures to determine if this could relate to oocyte degeneration or poor embryonic development [46]. They found that 22.2% of the time, the embryologist diverged from the recommended protocol, and there was an 8.5% damage rate [46]. Even more concerning was that in half of the oocytes which were inseminated using the correct technique, some type of oocyte anomaly was noted, namely in the cytoplasm or outer layer [46]. Fortunately, embryonic development was not impaired by these deviations. Yet, a small study showed a reduction in blastocyst formation rate in patients with sibling oocytes subjected to either ICSI or IVF with a history of MF or TFF (20% vs. 50%, *p* < 0.01), even when FR were similar, as were morphology on day two [47]. These studies highlight the concern for unnecessary risk without a benefit and concern for potential harm.

## 7. Conclusions

ICSI has provided an essential means to assist couples in having a family when severe MF is present. However, the original design and current utilization of ICSI are no longer in alignment. Over time ICSI has slowly become overused for NMF indications despite a lack of evidence of improvement in patient desired outcomes, specifically LBR per cycle. Additionally, clinics and/or physicians may be motivated to use ICSI for non-medical reasons, such as the availability of insurance or fear of poor outcomes, like TFF, without serious consideration of the potential adverse impact of its indiscriminate use. The use of ICSI is also presuming that TFF is due to sperm malfunction while not appreciating the complex nature of fertilization and embryo formation that go far beyond what ICSI can provide [48]. Finally, ICSI is not without potential harm of its own; this procedure has potential risks to oocytes and uncertain downstream effects on offspring. When applied indiscriminately, it uses valuable resources such as embryologists’ time and lab space, adding unnecessary costs, thereby taking it out of the financial reach and access of many without insurance, ultimately contributing to further disparities in care.

Recently three systematic reviews were published in 2020, all reporting either no difference in LBR between IVF/ICSI or a slight improvement with the use of IVF [49,50]. Yet, only Abbas reported on all three of the above-mentioned outcomes analyzed in this review: LBR, FR, and TFF showing improved FR with the use of IVF and no difference in TFF, all important outcomes for patients to consider [49]. However, this review dives further into potential differences in other outcomes to include the uncertainty in the health of offspring, potential damage to the oocyte, and the increase in demand of patient, provider, and embryologist’s cost/time. These are all important factors for all parties to consider when weighing the risks and benefits of IVF versus ICSI for the use of NMF. The authors do not hesitate to advocate for the benefits of ICSI in those clinical situations with severe MF or history of TFF; however, physicians and embryologists should critically assess the specific needs of each individual patient and utilize ICSI when appropriate indications are present.

## Figures and Tables

**Table 1:** ICSI use in NMF.

Author	Year	Study Design	FR	TFF	LBR
Khamsi [6]	2000	Review	IVF: 50.7–57.2% ICSI: 50–71.3% PN	IVF: 6.8–22.7% ICSI: 0% PN	NR
Bhattacharya [8]	2001	RCT	IVF: 61% ICSI: 50% NR	NR	NR
Khamsi [7]	2001	Prospective Controlled study	IVF: 57.2% ICSI: 71.3% *p* = 0.005	NR	NR
Tournaye [14]	2002	RCT	IVF: 59.6% ICSI: 67.6% NS	NR	NR
Kim [9]	2007	Prospective Controlled study	IVF: 69.1% ICSI: 69.1% *p* = 0.07	IVF: 3.2% ICSI: 4.0% *p* = 0.66	IVF: 22% ICSI: 17% *p* = 0.24
Butts [15]	2014	Retrospective Cohort	IVF: 21.9% ICSI: 20.4% *p* = 0.002	NR	NR
Kim [12]	2014	Retrospective Cohort	IVF: 79.1% ICSI: 83.4% *p* = 0.04	IVF: 0.6% ICSI: 4.2% NS	NR
Sfontouris [16]	2015	Retrospective Cohort	IVF: 65.3% ICSI: 58.7% NR	NR	IVF: 5.0% ICSI: 3.3% NS
Grimstad [17]	2016	Retrospective Cohort	IVF: 49.1% ICSI: 57.5% *p* < 0.0001	NR	IVF: 39.6% ICSI: 33.0% *p* < 0.0001
Schwarze [10]	2017	Retrospective Review	IVF: 73.55% ICSI: 73.84% *p* > 0.00	IVF: 3.37% ICSI: 4.49% *p* < 0.001	IVF: 28.76% ICSI: 22.99% *p* < 0.001
Tannus [18]	2017	Retrospective Cohort	IVF: 64% ICSI: 67% *p* = 0.25	IVF: 9.0% ICSI: 9.7% *p* = 0.73	IVF: 11.9% ICSI: 9.6% *p* = 0.71
Li [3]	2018	Retrospective Cohort	NR	NR	IVF: 39.2% ICSI: 36.2% NS
Liu [19]	2018	Retrospective Cohort	IVF: 61.56% ICSI: 76.00% *p* < 0.001	NR	IVF: 14.59% ICSI: 5.56% *p* < 0.05
Biliangady [20]	2019	Retrospective Cohort	IVF: 89.9% ICSI: 85.1% *p* < 0.001	NR	IVF: 32.71% ICSI: 24.26% *p* = 0.09
Drakopoulos [21]	2019	Retrospective Cohort	IVF: 66.6% ICSI: 66.6% *p* = 0.08	NR	IVF: 20% ICSI: 12.8% *p* = 0.06
Farhi [22]	2019	Retrospective Cohort	IVF: 50.1% ICSI: 71.0% *p* < 0.001	NR	NR
Sustar [11]	2019	Retrospective Review	IVF: 67.1% ICSI: 62.3% *p* < 0.0001	NR	IVF: 17.22% ICSI: 13.2% *p* = 0.001
Haas [23]	2020	RCT	IVF: 72.4% ICSI: 65.1% *p* = 0.38	NR	NR
Liu [24]	2020	Retrospective Cohort	NR	NR	IVF: 41.68% ICSI: 43.11% *p* = 0.599
Supramaniam [25]	2020	Retrospective Cohort	IVF: 64.7% ICSI: 67.2% *p* < 0.001	IVF: 17.3% ICSI: 17.0% *p* = 0.199	IVF: 12.2% ICSI: 12.4% NS
Isikoglu [13]	2021	RCT	IVF: 56.20% ICSI: 64.78% (per inseminated oocyte) NR	IVF: 15.63% ICSI: 0% NR	NR
McPherson [26]	2021	Retrospective Cohort	IVF: 31% ICSI: 14% *p* < 0.0001	NR	IVF: 94.3% ICSI: 92.4% NS

NR: Not reported; NS: Not significant; PN: *p*-value not reported; RR: Relative risk.
